# Highly Selective and Sensitive Self-Powered Glucose Sensor Based on Capacitor Circuit

**DOI:** 10.1038/s41598-017-01665-9

**Published:** 2017-05-03

**Authors:** Gymama Slaughter, Tanmay Kulkarni

**Affiliations:** University of Maryland Baltimore County and Bioelectronics Laboratory, Department of Computer Science and Electrical Engineering, Maryland, USA

## Abstract

Enzymatic glucose biosensors are being developed to incorporate nanoscale materials with the biological recognition elements to assist in the rapid and sensitive detection of glucose. Here we present a highly sensitive and selective glucose sensor based on capacitor circuit that is capable of selectively sensing glucose while simultaneously powering a small microelectronic device. Multi-walled carbon nanotubes (MWCNTs) is chemically modified with pyrroloquinoline quinone glucose dehydrogenase (PQQ-GDH) and bilirubin oxidase (BOD) at anode and cathode, respectively, in the biofuel cell arrangement. The input voltage (as low as 0.25 V) from the biofuel cell is converted to a stepped-up power and charged to the capacitor to the voltage of 1.8 V. The frequency of the charge/discharge cycle of the capacitor corresponded to the oxidation of glucose. The biofuel cell structure-based glucose sensor synergizes the advantages of both the glucose biosensor and biofuel cell. In addition, this glucose sensor favored a very high selectivity towards glucose in the presence of competing and non-competing analytes. It exhibited unprecedented sensitivity of 37.66 Hz/mM.cm^2^ and a linear range of 1 to 20 mM. This innovative self-powered glucose sensor opens new doors for implementation of biofuel cells and capacitor circuits for medical diagnosis and powering therapeutic devices.

## Introduction

Nanotechnology-based devices hold significant potential for improving the management of blood glucose levels in individuals suffering from diabetes by enabling highly sensitive and real-time monitoring of blood glucose. Such nano-biosensors can be used to emulate the body’s physiological needs to trigger the delivery of insulin to provide effective therapeutics for type 1 diabetes. An estimated 1.25 million American children and adult have type 1 diabetes and projections are that the U.S. will have approximately 587,000 children living with type 1 diabetes by 2050^1^. In type 1 diabetes, the insulin-secreting pancreatic islets cease to produce the 51-amino-acid peptide that is need for the regulation of blood glucose, thereby resulting in a deficiency in insulin and putting the patient at risk of hyperglycemia. The long term complications from hyperglycemia result in 2/3 of the costs of treating people with diabetes and lead to increased morbidity and mortality. Studies have shown that patients on intensive control programs who maintained their blood glucose levels close to normal experienced far less complications than patients who routinely maintained higher blood glucose levels^2, 3^. These conditions require periodic subcutaneous insulin injections to regulate the patient’s metabolism. At times, this can be painful, time-consuming and cumbersome, therefore, leading to poor patient compliance^4^. Studies have also suggested that some diabetics choose not to strive for close blood glucose control due to the intrusiveness of current blood sampling, assay methods and to maintain lower or more normal blood glucose levels puts them at an increased risk of hypoglycemia^5^. This situation is further exacerbated because blood sampling is relatively infrequent, compared to the rate of blood glucose fluctuations.

To overcome the drawbacks of discrete glucose sampling, we report a self-powered glucose sensor based capacitor circuit that provides the desired continuous blood glucose sensing. The system is based on the generation and accumulation of electrical power in a capacitor via a charge pump integrated circuit as a result of the oxidation of glucose. We reasoned that the frequency of charging/discharging the capacitor would be an ideal glucose sensing scheme, with improved detection sensitivity and selectivity without the use of a potentiostat circuit or an external power source as used in glucometers and continuous glucose monitors (CGMs). The self-powered glucose sensor setup allows system miniaturization to be easily accomplished as well as the construction of sensing arrays. We therefore designed and developed an innovative self-powered glucose sensor based capacitor circuit that comprises of an enzymatic glucose biofuel cell and capacitor for generating bioelectricity and sensing glucose, respectively.

## Results

### MWCNTs surface functionalization with 1-pyrenebutanoic succinimidyl ester and biocatalyst

**Figure 1:**
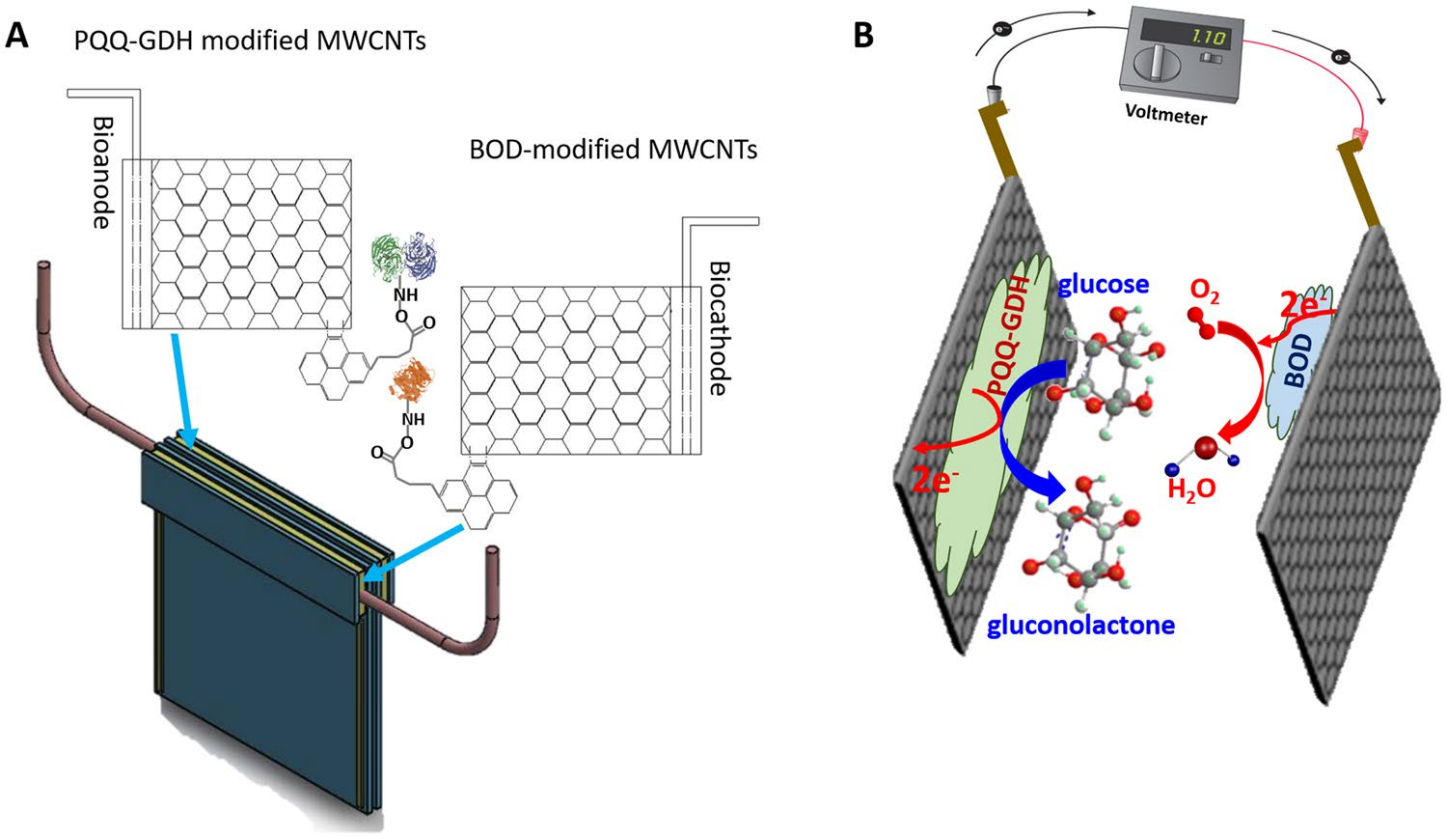
(**A**) Schematic illustration of the enzymatic glucose biofuel cell unit construction using dense mesh network of multi-walled carbon nanotubes (MWCNTs) modified with PQQ-GDH and BOD at the bioanode and biocathode, respectively; (**B**) 3D glucose biofuel cell unit and its function in the electrocatalytic oxidation of glucose and reduction of molecular oxygen.

To functionalize the surface of the bioelectrodes, we used a multi-step approach. Each bioelectrode surface is first treated with a heterobifunctional crosslinker, 1-pyrenebutanoic succinimidyl ester (PBSE, 1 mM) prepared in dimethyl sulfoxide (DMSO) to chemically crosslink the mesh dense network of MWCNTs via the π–π bonding^6^. Following the PBSE treatment, we covalently attached the glucose selective enzyme, pyrroloquinoline quinone glucose dehydrogenase (PQQ-GDH) dissolved in phosphate buffer saline (PBS) onto the PBSE modified MWCNTs for specific glucose binding. This protocol was repeated for the biocathode using the oxygen selective enzyme, bilirubin oxidase (BOD). All surface modifications were done by immersing the bioelectrodes in the respective reagents. The functionalized bioelectrodes show high specificity for glucose and oxygen binding. Furthermore, the bioelectrodes were coated with nafion to selectively screen against interfering analytes. Each bioelectrode employed in the biofuel cell has an active surface area of 0.04 cm^2^ and is formed from two mesh network of MWCNT substrate frames, and 200 μm tungsten wire is manually attached by silver conductive epoxy (Fig. 1A). This enables the fixture to be handled easily and provides for electrical connection to the recording instrument. The region around the uppermost edges of the substrate frames is sealed with polyimide HD-2611 to ensure good insulation.

Biofuel cell characterization in the presence of continuous supply of glucose analyte determines the bioelectricity parameters by analyzing its current-voltage response. The current-voltage response of the biofuel cell showed that successive changes in glucose concentration levels have a direct correlation with the current produced. The current increased immediately with increases in the glucose concentration levels, and it quickly reached a steady-state. The average response time of our biofuel cell was as short as 1 s (reaching 95% of steady state short circuit current). We designed the biofuel cell to achieve direct electron transfer between the active center of the enzyme and the current collector, where the electrons produced from the oxidation of glucose at the anode is transferred to the electrode and flows through the external circuitry to recombine at the cathode for the reduction of molecular oxygen to water (Fig. 1B). The transfer of electrons yielded a measurable current (1.325 mA/cm^2^) flow through the external circuitry. We further tuned the enzyme loading at the anode and cathode, thereby maximizing the sensing sensitivity. The optimal enzyme loading for the anode and cathode were 3 mg/ ml. For increased throughput analyses, we laid the biofuel cell unit down in a holder with a microfluidic chamber placed on top and connected to a peristaltic pump using a syringe. The chamber had a sample volume of 100 μL and enabled triplicate measurements under physiological conditions (37 °C and pH 7.4).

### MWCNT nanostructures enhance the power generation of enzymatic biofuel cells

**Figure 2:**
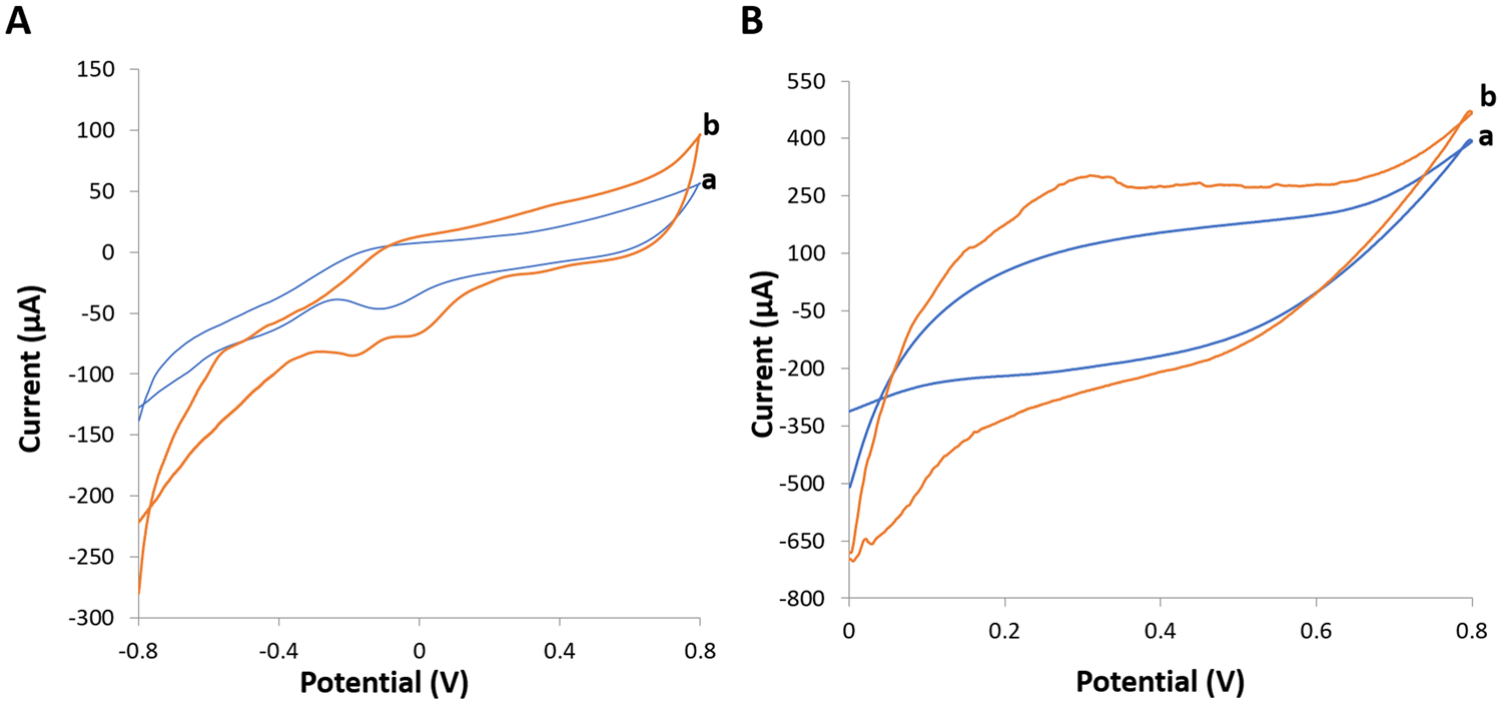
Cyclic voltammogram of the bioelectrodes. (**A**) Bioanode in the absence of glucose (a) and presence of 5 mM glucose (b). (**B**) Biocathode in 100 mM phosphate buffer saturated with air (a) and in the presence of O_2_ (b). All CVs were performed at 37 °C and pH 7.4.

**Figure 3:**
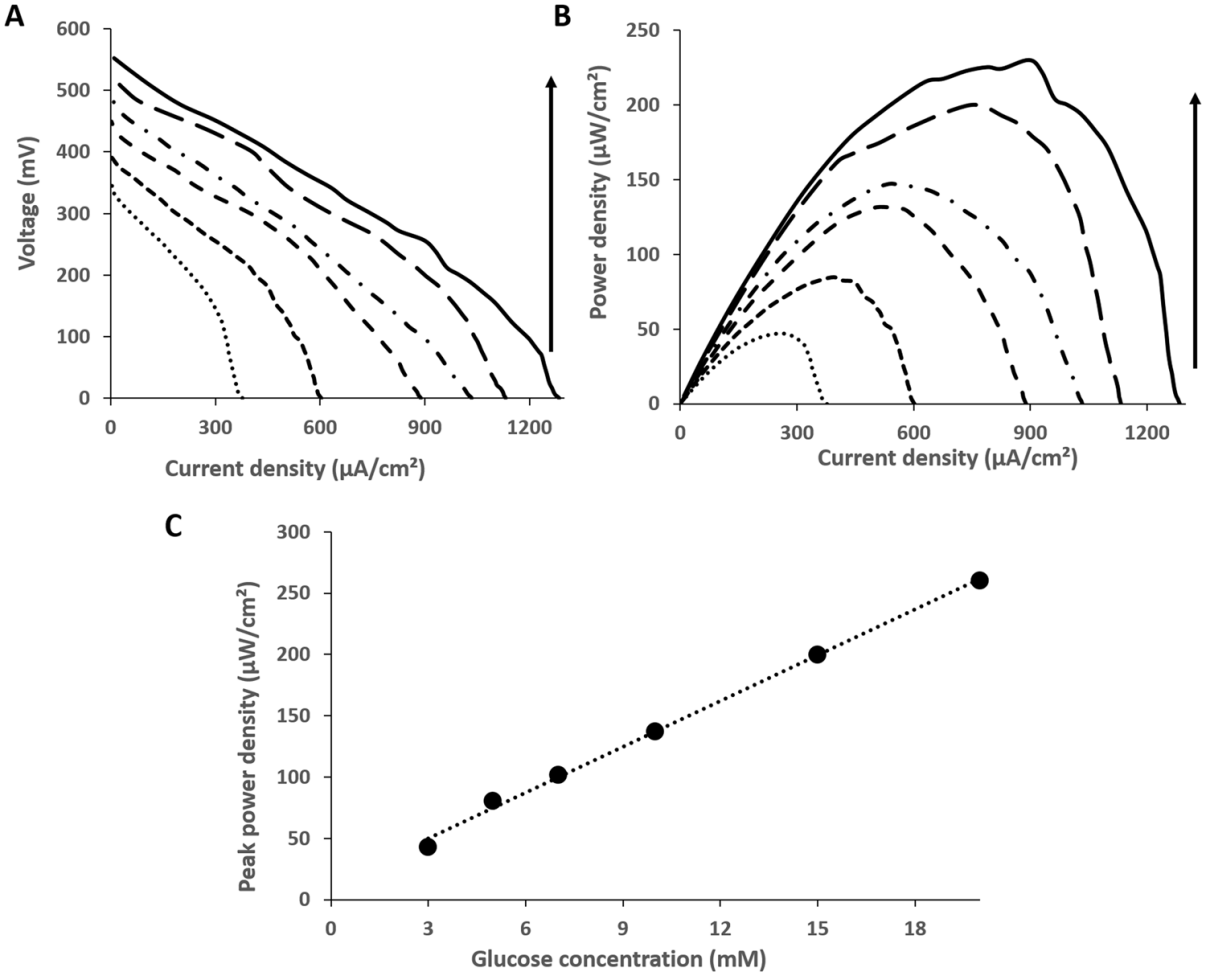
(**A**) Polarization characteristics of the enzymatic glucose biofuel cell composed of the PQQ-GDH modified buckypaper bioanode and the BOD modified buckypaper biocathode operating in increasing standard glucose solution (37 °C, pH 7.4 [3, 5, 7, 10, 15, 20 mM]); (**B**) The power curve as the functions of the current density; (**C**) Corresponding calibration curve of biofuel cell (peak power density vs. glucose concentration levels).

Figure 2 shows the typical electrocatalytic response of the bioanode and biocathode in the presence of 5 mM glucose and oxygen saturated PBS, respectively. The cyclic voltammogram (CV) confirmed the direct electron transfer between the active center of the bioelectrodes and the MWCNTs. The CV experiments were performed at physiologic conditions (37 °C, pH 7.4) at a scan rate of 10 mV s^−1^. The characteristic oxidizing onset voltage for PQQ-GDH in the presence of 5 mM glucose was detected at around −298 mV vs Ag/AgCl (Fig. 2A) which is higher than previously reported^7^, whereas the reducing onset voltage for bilirubin in the presence of oxygen was detected at 434 mV vs Ag/AgCl (Fig. 2B). To characterize the biofuel cell, we supplied various glucose concentrations continuously to the biofuel cell through the use of a peristatic pump and the current-voltage response was acquired using a variable load resistor. Following the acquisition of the current-voltage response, we calculated the current and power densities using the geometrical surface area of the active region of the bioelectrode (Fig. 3A). The polarization curves as a function of glucose concentration showed the electrical power density to be linearly related to the amount of glucose that was supplied to the biofuel cell (Fig. 3B). Therefore, both the open circuit voltage and the short circuit current generated by the biofuel cell was observed to be in correlation with glucose concentration. In 20 mM glucose, the open circuit voltage and short circuit current density along with a peak power density were 0.552 V, 1.285 mA/cm^2^ and 0.225 mW/cm^2^ at a cell voltage of 0.285 V, respectively. The observed open circuit voltage, short circuit current and power densities under physiological conditions (5 mM, pH 7.4 and 37 °C) were 0.391 V, 0.603 mA/cm^2^ and 84.64 μW/cm^2^ at a cell voltage of 0.214 V, respectively. These results are comparable and in some cases higher than previously reported values for biofuel cell bioelectrodes based on mesh network of MWCNTs, carbon cavity microelectrodes, and carbon fibers listed in Table 1 ^8–12^. Compared with these biofuel cells, the performance of our biofuel cell configuration was superior because of its fast response time (~1 s) and high sensitivity (311.75 μW/mM.cm^2^) and peak power density (0.225 mW/cm^2^). Note that this glucose biofuel cell presented excellent linear peak power-concentration relationship at the concentration regime ranging from 3 to 20 mM (Fig. 3C). The linear coefficient was calculated to be 0.997 in this regime. Therefore, the biofuel cell as a standalone power source device can function as a glucose sensor using glucose selective enzymes to oxidize glucose^13, 14^.

**Table 1:** Open circuit (V_oc_), short circuit current (I_sc_) and power density (P_density_) values collected from refs 8–12.

Electrode	Anode	Cathode	Fuel	V_OC_ (mV)	I_SC_ (µA)	P_density_ (µW/cm^2^)	Reference
Buckypape	PQQ/GDH	Laccase	Glucose	524	13.5	43.3	8
Flexible Carbon fiber	GO_x_/BSA/Gt/NR	PAMAM/PtNPs	Glucose	400	0.27	200	9
Carbon Rods	Trehalaselglucose oxidase trehalose	Bilirubin Oxidase	Trehalose	450	1840	100	10
Carbon Cavity	Go_x_/phenanthrenequinone	Tetrathiafulvalene/PPO	Glucose	320	~78	40.8	11
Buckypaper	PQQ/GDH	Bilirubin oxidase	Glucose	480.1	25.6	89.04	12

### Enzymatic biofuel cells have biosensing characteristics

**Figure 4:**
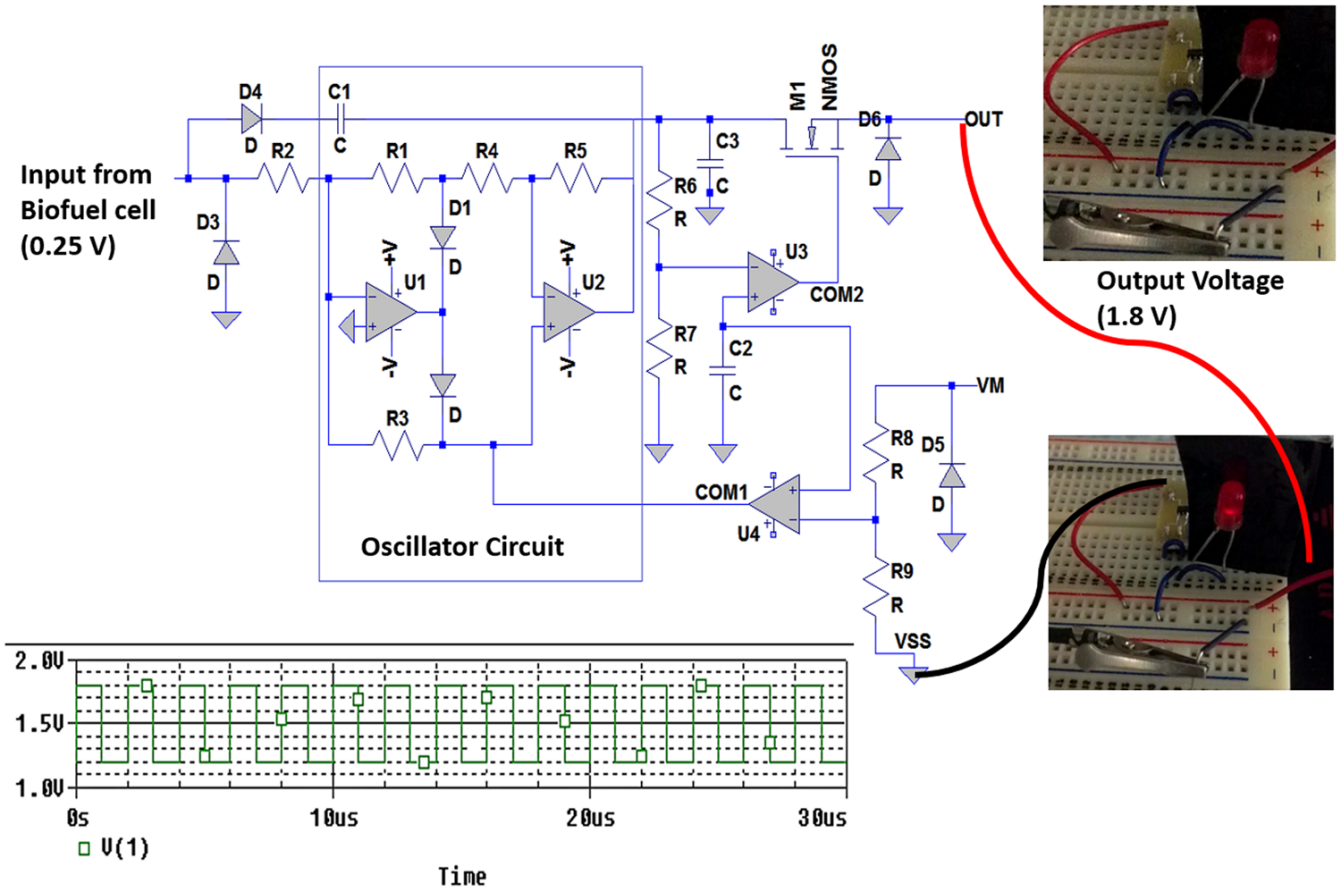
Schematic diagram of the charge pump circuit. The input electrical power from the enzymatic glucose biofuel cell drives the charge pump circuit, wherein the voltage is amplified and charged into the capacitor until it reaches 1.8 V. After which the circuit switch discharges the capacitor and enables current to be supply to the light emitting diode (LED) as a pulsed wave voltage between 1.2 to 1.8 V.

Rapid, amplification of the power generated by the biofuel cell could improve powering of implantable bioelectronic devices and glucose monitoring. We integrated the biofuel cell with a charge pump and capacitor circuits. The charge pump circuit consists of capacitors and switches and can be modified per applications^15^. It requires a minimum input supply of 0.25 V from the biofuel cell to trigger its internal oscillation circuit as shown in Fig. 4. As such, the clock signal from the oscillation circuit steps up the input voltage from the biofuel cell and gradually charges to start up the capacitor circuit interfaced to the charge pump circuit. Once the voltage across the capacitor reaches the threshold voltage (1.8 V), the charge pump circuit switch discharges the capacitor to the stop discharge voltage of 1.2 V from the OUT pin and the electric power is charged to the capacitor all over again. We used the charge pump circuit to amplify the nominal biofuel cell voltage to high voltage (1.8 V) along with high current (1.46 mA) using a 0.1 μF capacitor, which then supplied that stored energy as a burst of power sufficient enough to drive a light emitting diode (LED) without affecting the design of the biofuel cell. This demonstrates the ability of a single biofuel cell to power a small electronic device. The rate at which the electrical power is supplied via the charge pump circuit is dependent on the concentration of glucose and the capacitance of the capacitor. As the capacitance of capacitor is increased, the frequencies of the charge/discharge cycle decreases. Additionally, the resulting charge/discharge voltage can be observed across the capacitor, which in turn serves as the transducer.

**Figure 5:**
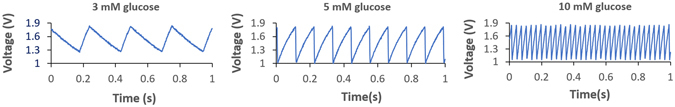
Effect of glucose concentration levels on the charging/discharging frequency of the capacitor connected to the charge pump operating in the presence of 3 mM, 5 mM and 10 mM glucose.

Self-powered glucose sensor studies revealed the effect of glucose concentration on the charge/discharge frequency of the capacitor circuit to be dependent on the capacitance of the capacitor and the electrical potential, current and power generated by the biofuel cell. This in turn is dependent on glucose concentration, since glucose oxidation is catalyzed by PQQ-GDH at the anode increases in glucose concentration-dependent manner, thereby enabling the capacitor to function as a transducer (Fig. 5).

We further tuned the glucose concentration levels, thereby maximizing the linear dynamic range (1–45 mM) and detection sensitivity (23.12 Hz/mM.cm^2^). The charge/discharge frequency of the capacitor correlated well (R^2^ = 99.81%) with increasing glucose concentration levels:

1 $$Frequency\,response\,(Hz)=0.9252\,[glucose]\,(mM)+14.113\,(Hz)$$

### pH and temperature dependent studies

**Figure 6:**
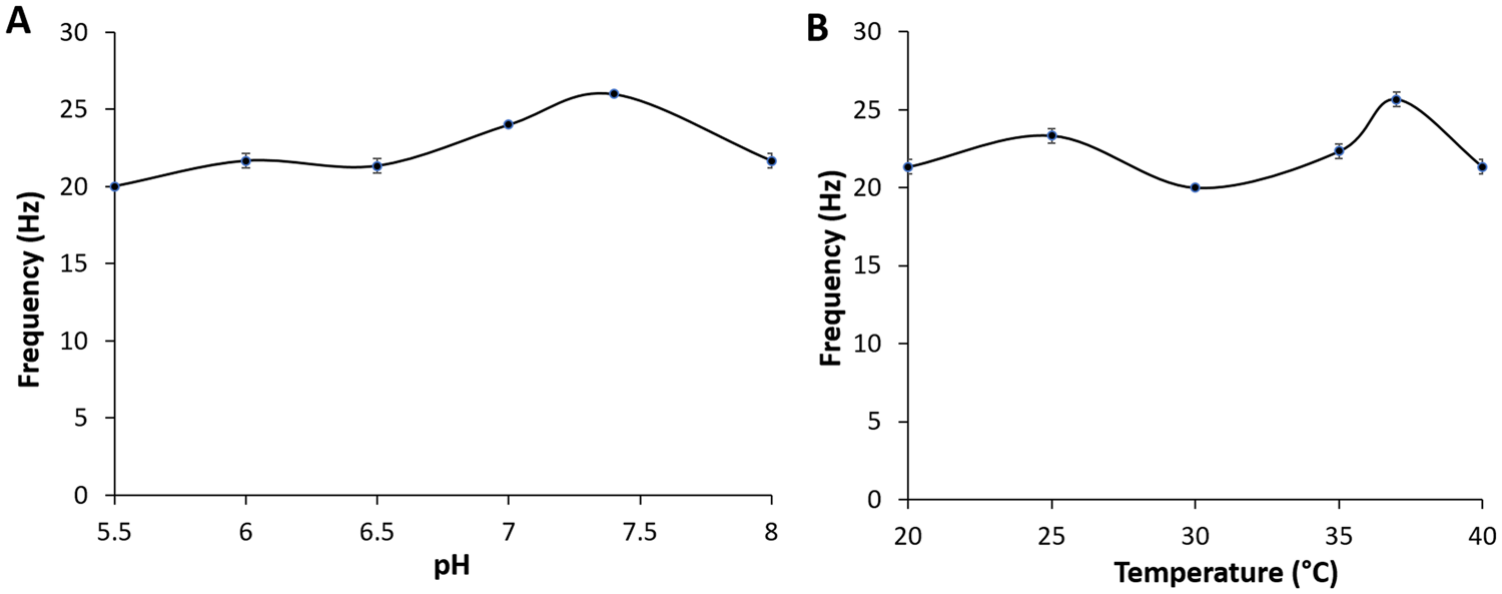
(**A**) Effect of pH on response of the enzymatic glucose biofuel cell at 37 °C in 7 mM glucose; (**B**) Response curve of the enzymatic glucose biofuel cell to 7 mM glucose under various temperatures at 37 °C, pH 7.4.

To establish pH and temperature dependent profiles in response to glucose for the present self-powered glucose sensor, we first exposed the system to 7 mM glucose over the investigated pH range of 5.5–8.0 (Fig. 6A). The observed optimal pH was 7.4, which was significantly higher than that of system based on laccase as the cathodic enzyme (~7)^16^. Such optimal pH of the self-powered glucose sensor is attributed to the use of BOD as the cathodic enzyme, thereby enabling this system to operate at physiologic pH as this is essential from *in vivo* and *ex vivo* perspective. We next investigated the temperature range of 20–40 °C (Fig. 6B). With respect to the temperature profile, the response intensity of the self-powered glucose sensor increased with temperature from 30–37 °C and subsequently decreased at 40 °C. We observed two optimal temperatures of 25 °C and 37 °C, which indicates that the system is thermally stable at room temperature as well as at physiological temperature. Additionally, the 37 °C optimal represents about a 3 °C shift when compared to the 40 °C optimum observed for the laccase based system^16^. The observed 37 °C optimum for this system is ideal for *in vivo* and *ex vivo* applications.

### Self-powered glucose sensor based on capacitor circuit can selectively screen against interfering analyte

To determine the device selectivity, we monitored the charge/discharge frequency of the capacitor in the self-powered glucose sensor in the presence of competing and non-competing analytes under physiological concentrations close to *in vivo* environment. The physiological glucose concentration in serum is ~5 mM whereas the physiological concentration of competing (i.e., fructose, maltose and galactose) and non-competing (i.e., ascorbic acid and uric acid) interfering species is ≤0.42 mM^17^. These interfering species have been shown to have a significant impact on the performance of the glucose sensors^18, 19^. The self-powered glucose sensor based on capacitor circuit showed no response to 0.2 mM ascorbic acid or 0.5 mM uric acid in the presence of 5 mM glucose. This could be attributed to the low voltage (<0.6 V) generated by the sensor system, which is well below the 0.6 V to 0.7 V vs. Ag|AgCl|KCl_sat_ necessary for the decomposition of ascorbic and uric acid^20^. Moreover, the bioelectrodes were coated with a negatively charged polymer, Nafion, which prohibits the passage of negatively charged interfering species, such as ascorbic and uric acid, to the bioelectrodes^21^. The ability of the Nafion membrane to restrict the penetration of non-competing analytes in complex medium improved the accuracy of the self-powered glucose sensor. Interestingly, no response was observed for fructose, maltose or galactose in the presence of 5 mM glucose. However, a negligible voltage response to these three competing analytes was observed when introduced individually. This negligible voltage was insufficient to drive the charge pump circuit, thereby no charge/discharge frequency could be observed across the capacitor. Thus, both competing and non-competing analytes at their physiological concentration have no effect on the performance of the self-powered glucose sensor based on capacitor circuit. The self-powered glucose sensor exhibits unprecedented selectivity to quantitatively screen against interfering species.

### Stability of electrical power generation by self-powered glucose sensor

**Figure 7:**
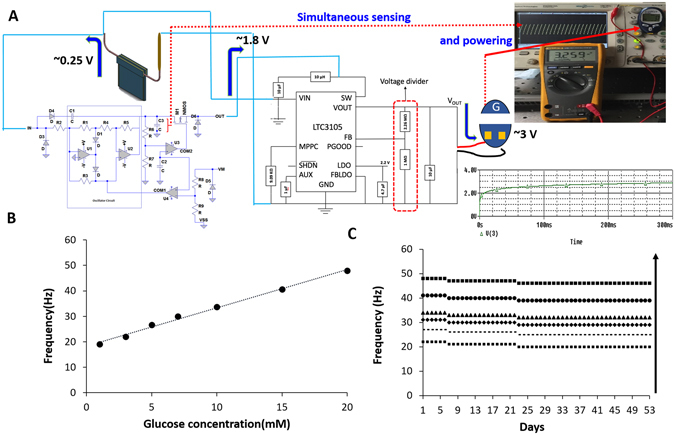
(**A**) Circuit block diagrams of the charge pump circuit interfaced to the step-up DC convertor and its application in powering a LifeStyle glucometer within 300 ms while sensing glucose via the charging/discharging frequency of the capacitor; (**B**) Dependence of the charging/discharging frequency of the capacitor on glucose concentration, as measured with the self-powered glucose biosensing system [1, 3, 5, 7, 10, 15, 20 mM]; (**C**) Operational stability response of the self-powered glucose biosensing system in various glucose concentrations [3, 5, 7, 10, 15, 20 mM].

Several technical modifications could be made to improve the self-powered glucose sensor with capacitor circuit and accelerate its application for continuous glucose sensing and powering bioelectronics devices (i.e., glucometer). First, using a step-up DC converter circuit, we converted the burst supply of power from the self-powered glucose sensor into a steady DC output supply that is substantially higher (3.2 V) for powering small devices. This converter circuit requires a triggering voltage of 1.4 V to start its operation and maintains its operation with a minimum operating voltage of 0.225 V, all of which is easily fulfilled by the self-powered glucose sensor. Second, we implemented the step up DC converter circuit using a voltage divider circuit as illustrated in Fig. 7A to tune the DC output voltage by varying the R_1_ and R_2_ ratio in the following equation:

2 $${{\rm{V}}}_{{\rm{OUT}}}=1.004\ast ((\frac{{{\rm{R}}}_{1}}{{{\rm{R}}}_{2}})+1)$$

PSpice simulation studies of the above circuit revealed that the output voltage increases with the increase in the resistance ratio according to ohm’s law. However, the experimental implementation showed that the output voltage varies from those obtained during the simulation. This could be attributed to various voltage losses arising from the circuit design that was not considered in the simulation. The resulting experimental resistance ratio of 1.390 Ω, 1.632 Ω, and 2.260 Ω yielded steady output voltages of 1.95 V, 2.07 V, and 3.2 V, respectively. The steady 3.2 V_DC_ output voltage achieved with R_1_ = 2.26 MΩ and R_2_ = 1 MΩ was sufficient to power an LED, digital thermometer and glucometer within 300 ms as illustrated in Fig. 6A.

This novel self-powered glucose sensor system continuously sensed glucose while simultaneously operating a FeeStyle Lite glucometer, which requires a 3 V input supply. The calibration curve of the system presented in Fig. 7B showed a dynamic range of 1–20 mM glucose with the following regression equation (R^2^ = 0.9942):

3 $$Frequency\,response\,(Hz)=1.5062\,[glu{cose}]\,(mM)+(18.302)\,(Hz)$$

which is broad enough for most blood samples in clinical applications. Since glucose sensing was dependent on the power generated by the self-powered glucose sensor, the charge/discharge frequency was slightly enhanced by the simultaneous operation of the glucometer. This resulted in an increased sensitivity to 37.66 Hz/mM.cm^2^, which is over 2-fold higher than previously reported^13, 22^. These results demonstrated that by monitoring the frequency of the capacitor’s charge/discharge voltage, glucose concentration levels can be measured while simultaneously powering a small electronic device via the OUT pin.

To demonstrate that the self-powered glucose sensor is stable under continuous operation, we performed the concentration-varying experiment in which concentration of glucose was successfully increased from 3 mM to 20 mM concentration under constant load discharge for 1 h each day over a period of 53 days, while monitoring the charge/discharge frequency of the capacitor (Fig. 7C). A slight drop of 2.08% to 4.54% in the charge/discharge frequency was observed in the presence of 20 mM to 3 mM glucose after the first week of continuously operation. The overall drop in the frequency was 4.17% to 9.09% in the presence of 20 mM to 3 mM glucose over a period of 53 days of operation. The device presented almost the same signals for the same glucose concentrations over the 53 days and retained greater than 91% of its activity at day 53. This stability was in sharp contrast to the self-powered glucose sensing system based on bulky potentiostat circuit^23^ and glucose biofuel cell that exhibited a 7-day stability and retained less than 41% of its activity^24^. This self-powered glucose sensor stability is greatly enhanced by the capacitor’s capacitance, thereby enabling stable continuous monitoring of glucose concentration.

## Discussion

Based on our findings, we explored the biofuel cell bioelectricity generation to power a small electronic device via a charge pump circuit and a step-up DC converter circuit while simultaneously monitoring glucose concentration levels. Although the single biofuel cell shows potential to replace batteries as a power source, the electrical power generated must be amplified as it is neither sufficient to sense glucose nor power microelectronic devices. Previous work has shown that the micropower generated can be improved by stacking multiple biofuel cells in series or parallel depending on the applications. However, this makes the overall design bulky and increases the circuit complexity^13, 23, 25^. By integrating the biofuel cell with a charge pump and capacitor circuits, we created a highly portable platform capable of both power amplification and rapid sensing of glucose concentration levels from the single biofuel cell. We established a quantitative protocol that reports both power characteristics and glucose concentration levels, while powering small electronic device.

Unlike conventional amperometric glucose sensors that require bulky potentiostat circuit, the self-powered glucose sensor based on capacitor circuit employed dense mesh network of MWCNTs, which provided an enlarged specific surfaces for enzyme immobilization and facilitate the transport of glucose molecules, hydrogen ions and electrons. This scheme made it possible to use a compact setup and construct densely packed sensing unit that exhibited unprecedented sensitivity^16^ and linear dynamic range^26^. This nanoscale platform offered a much greater effective surface area than planer materials^27^, which is critical for increasing the density of immobilized enzyme, and hence the sensitivity of the self-powered glucose sensors. The fast response of our biofuel cell was attributed to the porous MWCNT nanostructures that facilitated the diffusion of the glucose.

The high sensitivity resulted from the immobilization of the enzyme via PBSE on the MWCNTs and the Nafion coating, which selectively screened against interfering analytes, preserved the high activity of the enzyme and its catalytic effects. Additionally, both competing and non-competing analytes at their physiological concentration were shown to have no effect on the performance of the self-powered glucose sensor based on capacitor circuit. The self-powered glucose sensor exhibits unprecedented selectivity to quantitatively screen against interfering species. With increasing glucose concentration, the capacitor transducer displays charge/discharge frequency changes proportional to the target glucose levels. Compared to conventional glucose sensors (glucometers and CGMs), the self-powered glucose sensor technology offers highly sensitive and selective glucose analyses and enables continuous and dynamic monitoring of glucose levels in real-time, without the associated infrequent sampling of glucometers and frequent maintenance and replacement of continuous glucose monitors^28, 29^. In order to improve the power output of the system, we further integrated a DC-DC converter to enable the system to supply stable 3.2 V to power an external device, such as a glucometer. Therefore, the integrated system can be readily used to extract glucose concentration information from the biofuel cell, for glucose monitoring.

For the first time, a novel self-powered glucose sensor based on capacitor circuit that is capable of simultaneously sensing glucose and powering a digital device, such as a glucometer is demonstrated. The sensor system generates electrical power via the direct conversion of biomolecular recognition and binding events to electronic signals that can be monitor electronically. By combining the advantages of porous MWCNTs and energy amplification circuits, the sensor system exhibited unprecedented performance with high sensitivity, selectivity, and fast response time. Together, the approaches developed here will facilitate system miniaturization by yielding comprehensive glucose assays and electrical power information. This system is readily scalable for powering small electronic devices. We further envision that the self-powered glucose sensor system could be greatly reduced in footprint by using microsystem techniques and other inexpensive deposition methods to deposit dense mesh network of carbon nanotubes and metal wire traces. Additionally, the self-powered glucose sensor system along with the power amplification circuits could be eventually studied *in vivo*. Since the *in vivo* environment can be considered as unforgiving for any electronic devices, the power amplification circuits would have to be hermetically sealed in order to protect it from the *in vivo* environment. Although the self-powered glucose biosensor would continuously harness the biochemical energy of glucose as a fuel, the *in vivo* glucose homeostasis should not be significantly impacted as illustrated with other implantable technologies such as the continuous glucose monitor equipped with implantable glucose needle sensor. We believe that this type of high-performance, self-powered glucose sensor system, combined with low-cost construction shows great potential for use in biotechnology applications relating to medical diagnosis and diabetes management.

## Methods

### Materials and components

Buckypaper, a highly conductive material^30, 31^ consisting of aggregated chain of multi-walled carbon nanotubes (MWCNTs) were used as the base substrate material for enzyme immobilization and was purchased from Nanotech labs (Yadkinville, NC). 1-Pyrenebutanoic succinimidyl ester (PBSE) used as a bi-functional crosslinker between the base substrate and the enzyme was purchased from AnaSpec. Inc. Glucose selective enzyme, PQQ-GDH was purchased from Toyobo. Co. Ltd. Meanwhile, oxygen reducing enzyme bilirubin oxidase derived from Myrothecium was purchased from Sigma Aldrich. All other solvents such as dimethyl sulfoxide (DMSO) and potassium phosphate, D- (+)-Glucose, calcium chloride were purchased from Sigma Aldrich. Nafion® used to selectively screen against non-competing analytes was purchased from Sigma Aldrich^29^. The S882z charge pump circuit and LTC3105 step up DC converter were purchased from Seiko electronics and Linear technology, respectively.

### Electrode preparation

Two 500 μm × 500 μm square dense mesh network of MWCNTs were cut followed by sandwiching a ‘L’ shaped tungsten wire via silver epoxy between the square platforms to serve as the anodic substrate frame. The cathodic substrate frame was prepared in the same manner. Polyimide HD-2611 from HD Microsystems was used to insulate and seal the upper most region of the frames. The samples were then cured at 150 °C. The active surface area of the electrodes was designed to be 400 μm^2^. The tungsten wire was used to enable the delicate samples to be handled.

### Surface modification

Each of the electrode frames were rinsed with isopropyl alcohol (IPA) to remove surface impurities on the active region of the electrode. A heterobifunctional crosslinker, PBSE (1 mM), was used to chemically crosslink the MWCNTs via the ‘π–π’ bonding and form amide bond with the enzyme. Anodic enzyme, PQQ-GDH (1 mg/ml) was dissolved in 10 mM PBS (pH 7.0) +1 mM CaCl_2_ (pH 7.0) and cathodic enzyme, BOD was used to prepare bioanode and biocathode, respectively. The amine group from the enzymes replaces the ester group on PBSE, thereby resulting the immobilization of enzyme on the bioelectrodes^32^. These bioelectrodes were further coated with Nafion® to selectively screen against interfering analytes. The resulting bioelectrodes were used to construct the biofuel cell.

### Printed circuit board fabrication

Eagle computer aided design (CAD) tool was used to design the printed circuit board (PCB) layout for the charge pump and step up DC converter circuits, which can store the electrical power generated from the biofuel cell and the capacitor circuit and release the energy when triggered. The board layouts were printed after the design was cleared of all the errors. Further, the distance between the pins of the integrated circuit and the output terminal were optimized to be as short as possible to avoid any signal losses. The PCB was fabricated using standard photolithography and circuit components were soldered onto the PCB to complete the PCB fabrication process.
